# Use of the painDETECT to discriminate musculoskeletal pain phenotypes

**DOI:** 10.1186/s40945-022-00129-2

**Published:** 2022-02-17

**Authors:** Juliana Valentim Bittencourt, Márcia Cliton Bezerra, Mônica Rotondo Pina, Felipe José Jandre Reis, Arthur de Sá Ferreira, Leandro Alberto Calazans Nogueira

**Affiliations:** 1 Rehabilitation Science Postgraduate Program at Augusto Motta University Centre (UNISUAM), Paris, 84, Bonsucesso, Rio de Janeiro, RJ CEP 21041-020 Brazil; 2 Physiotherapy Department at Federal Institute of Rio de Janeiro (IFRJ), Rio de Janeiro, Brazil

**Keywords:** Musculoskeletal pain, Neuropathic pain, Pain mechanisms, Central nervous system sensitization, Diffuse noxious inhibitory control

## Abstract

**Background:**

Musculoskeletal pain patients present similar pain characteristics regardless of the clinical diagnosis. PainDETECT questionnaire is useful for screening neuropathic-like symptoms in many musculoskeletal conditions. However, no previous studies compared pain phenotypes of patients with musculoskeletal pain using the painDETECT. Therefore, the current study aimed to compare the pain characteristics of patients with musculoskeletal pain classified according to the painDETECT as nociceptive pain, unclear, and neuropathic-like symptoms.

**Methods:**

A cross-sectional study was conducted in 308 participants with musculoskeletal pain. Demographic and clinical characteristics of the participants were examined. Neuropathic-like symptoms, pain intensity, pain area, Central Sensitization-related sign and symptoms, functional limitation, and conditioned pain modulation were assessed in patients with musculoskeletal pain. Independent one-way analysis of variance (ANOVA) was used to test for between-group differences for the outcome measures with continuous variables and Pearson chi-square test verified between-group differences on the efficiency of the conditioned pain modulation.

**Results:**

Participants had a mean age of 52.21 (±15.01) years old and 220 (71.42%) were females. One hundred seventy-three (56.16%) participants present nociceptive pain, 69 (22.40%) unclear, and 66 (21.42%) neuropathic-like symptoms. A one-way ANOVA showed differences for the pain intensity [F (2,305) = 20.097; *p* < .001], pain area [F (2,305) = 28.525; *p* < .001], Central Sensitization-related sign and symptoms [F (2,305) = 54.186; *p* < .001], and functional limitation [F (2,256) = 8.061; *p* < .001]. However, conditioned pain modulation was similarly impaired among the three groups (*X*^2^ = 0.333, *p* = 0.847).

**Conclusion:**

Patients with neuropathic-like symptoms revealed unfavorable pain characteristics compared to their counterparts, including pain intensity, generalized pain, Central Sensitization-related sign and symptoms, and functional limitation.

## Background

Musculoskeletal conditions represent a common cause of pain and disability in the world population. In Europe, the United States, and Brazil approximately half of the population is affected by noninflammatory musculoskeletal pain [1–3]. Musculoskeletal pain patients may present similar pain characteristics regardless of the clinical diagnosis. Although musculoskeletal pain represents a heterogenous group, musculoskeletal conditions from different anatomic sites share similar pain characteristics [4]. Moreover, five musculoskeletal pain phenotypes were described independent of primary pain location [5]. Thus, classify musculoskeletal pain patients can be a challenge for health professionals.

Nociceptive and neuropathic pain are commonly reported by patients with musculoskeletal pain. Some musculoskeletal pain conditions classified as nociceptive pain (i.e., knee osteoarthritis [6], rotator cuff tears [7], and impingement syndrome of the shoulder [8]) may present neuropathic-like symptoms. Although there is an exchange of several pain characteristics that classify the predominance of nociceptive pain or neuropathic-like symptoms, previous studies showed that neuropathic-like symptoms patients had unfavorable outcomes [9–13]. For instance, increased pain and disability, low quality of life, and increased use of health resources are more reported by patients with low back pain radiating to the leg than in patients with low back pain alone [9]. Also, other studies reported more severe pain, poorer physical health, symptoms of depression, and psychological distress in neuropathic-like symptoms when compared to patients with nociceptive pain [10–13]. Therefore, it is essential to ascertain the divergences of pain characteristics present in these musculoskeletal pain conditions.

Several instruments have been used in the evaluation of patients with musculoskeletal pain. Central Sensitization Inventory (CSI) is the most used questionnaire for identifying Central Sensitization (CS)-related sign and symptoms [14]. Patients with knee osteoarthritis, which is regularly considered nociceptive pain, present CS-related sign and symptoms [15]. Likewise, CS-related signs and symptoms were reported in painful conditions with the neuropathic component [16, 17]. CSI scores have been related to pain intensity and pain area measured by Widespread Pain Index [18]. The impairment of the conditioned pain modulation (CPM) has been reported in patients with musculoskeletal pain [19, 20], chronic pain [21], and chronic widespread back pain and fibromyalgia syndrome [22]. Also, our group found a prevalence of 20% [23] and 25% [24] impaired CPM in patients with musculoskeletal pain.

PainDETECT questionnaire have been used in a large number of musculoskeletal conditions (low back pain, rheumatoid arthritis, osteoarthritis, cancer pain, and lumbar spondylolisthesis) [25]. PainDETECT is one of the best options for screening neuropathic-like symptoms (sensitivity = 85% and specificity = 95%) [26]. Moreover, the original version in German of the painDETECT presented an adequate internal consistency (Cronbach’s α = 0.76). Similarly, the Brazilian version of the painDETECT obtained an adequate internal consistency for the nine items (Cronbach’s α of 0.74) and for the seven symptoms of sensory pain (Cronbach’s α of 0.83, 27). Finally, painDETECT is a low-cost and simple screening instrument that may provide insight to the health professionals in the assessment and, consequently, the offer of strategies adequate for managing musculoskeletal pain patients. Therefore, the present study aimed to compare the pain characteristics of patients with musculoskeletal pain classified as nociceptive pain, unclear, and neuropathic-like symptoms according to painDETECT questionnaire. We hypothesized that patients with neuropathic-like symptoms have unfavorable clinical features compared to patients with nociceptive pain and unclear.

## Methods

### Study design and ethical considerations

A cross-sectional study design reported following the STrengthening the Reporting of OBservational studies in Epidemiology (STROBE) requirements [27]. This study was approved by the Research Ethics Committee of Augusto Motta University Centre (number: 03870618.5.0000.5235), in accordance with the Helsinki Declaration for research in humans. All patients who met the eligibility criteria signed the informed consent form before the study procedures.

### Study patients

Consecutive patients with musculoskeletal pain (aged 18 years and over) from two outpatient Physical Therapy departments (Gaffrée and Guinle University Hospital and Augusto Motta University Center), two private clinics, and an outpatient multidisciplinary rehabilitation department (Cabo Frio Rehabilitation Center) in Rio de Janeiro State, Brazil, were enrolled when they sought treatment between March and September 2019. The study included patients with acute pain (pain duration less than 3 months) and chronic pain (pain duration greater than 3 months). Musculoskeletal pain was defined as pain perceived in a region of the body with muscular, ligament, bone, or joint origin [2]. Patients who had a surgical procedure in the spine, pregnant women, patients with rheumatologic diagnosis in the acute inflammatory phase, tumors, being illiterate, or could not complete the self-reported questionnaires were excluded from the study.

### Procedures

Patients were referred for an initial evaluation consisting of the clinical history and physical examination. Data collection on sociodemographic (age, sex, weight, height, education level, and income) and pain characteristics (pain intensity, pain duration, CS-related sign and symptoms, and pain area) were performed by using a standard questionnaire. Neuropathic-like symptoms was measured by painDETECT questionnaire. Pain intensity was measured using the Numeric Pain Rating Scale from 0 to 10 (i.e., 0 is no pain, and 10 is the worst pain possible). Pain duration was recorded in months. CS-related sign and symptoms was assessed by Central Sensitization Inventory (CSI). Generalized pain was evaluated by Widespread Pain Index. Conditioned Pain Modulation (CPM) was assessed by Cold Pressor Test. Finally, functional limitation was measured using the Patient-Specific Functional Scale. The completion of all questionnaires was supervised by an examiner for clarification in case of uncertainties and lasted approximately 10 min per participant. After completing questionnaires, patients were referred for evaluation of CPM on the same day.

painDETECT questionnaire *–* The painDETECT is a self-administered questionnaire that encompasses four domains as follows: intensity of the pain (three questions), pain course pattern (four graphs), areas of pain and the presence of radiating pain (body chart drawing), and sensory descriptor items of pain (seven questions). The first domain presents three questions regarding pain intensity at the moment, the strongest pain level (last 4 weeks), and pain level on average (last 4 weeks). The final score is calculated by nine-item represented in the last three domains (pain course pattern, radiating pain, and gradation of pain). The score of the second domain (pain course pattern) varies between 0 or + 1, and the answer options are: Persistent pain with slight fluctuations = 0; Persistent pain with pain attacks = − 1; Pain attacks without pain between them = + 1; and Pain attacks with pain between them = + 1. The third domain (radiating pain) has the question: “Does your pain radiate to other regions of your body?”. The answer to this question is dichotomous (yes/no) and varies between + 2 / 0. The fourth domain (gradation of pain) have seven questions with six possible answers for each question scoring from 0 (never); 1 (hardly noticed); 2 (slightly); 3 (moderately); 4 (strongly); to 5 (very strongly). A final score between − 1 to 38 can be achieved by summing up the scores given in each domain. The painDETECT is validated for a large number of neuropathic pain conditions [28–30]. It was also validated for use in mixed pain conditions such as rheumatoid arthritis, osteoarthritis, cancer pain, and lumbar spondylolisthesis [25]. The cut-off points for the original questionnaire indicate that a neuropathic component is unlikely in the scores ≤12, a neuropathic component is unclear in scores between 13 and 18, whereas values ≥19 a neuropathic component is probable [25]. For screening purposes, we considered scores ≤12 as nociceptive pain, scores between 13 and 18 as unclear, and scores ≥19 as neuropathic pain. The painDETECT questionnaire was adapted cross-culturally to the Brazilian context [31].

### Outcome measures

Pain intensity was measured during the initial evaluation using the Numeric Pain Rating Scale from 0 (no pain) to 10 (worst pain possible). Participants were oriented to rate their pain intensity at the moment of the initial evaluation. The duration of pain was recorded in months, and patients were classified with chronic musculoskeletal pain if they had pain for more than 3 months, according to the International Association for the Study of Pain [32].

CS-related sign and symptoms – The Central Sensitization Inventory (CSI) identified patients whose presentation symptoms may be related to central sensitization. CSI is an instrument developed to identify CS-related sign and symptoms [33]. Part A assesses 25 health-related symptoms commonly observed in patients with central sensitivity syndrome and is scored on a 5-point Likert scale from 0 (never) to 4 (always), with a total of 100 points. Higher scores represent an increase in the severity of symptoms. Part B is not scored and encompasses ten previous diagnoses of an individual, including seven central sensitivity syndromes and three disorders related to central sensitization syndrome. The optimal cut off point was established at 40/100 in patients with central sensitivity syndrome [34, 35]. Also, the severity of CS-related sign and symptoms has been classified into sub-clinical (0–29), mild [29, 30, 32–39], moderate [40–49], severe [50–59] and extreme (60–100), where higher scores indicate an increase in the severity of symptoms. The Brazilian version of the CSI demonstrated strong psychometric properties [36].

Pain area – Widespread Pain Index was used to diagnose generalized pain. The Widespread Pain Index is composed of a list of 19 body areas shoulder girdle, upper arm, lower arm, hip, upper leg, lower leg, and jaw in all these areas are considered left and right. The areas chest, abdomen, upper back, lower back, and neck also composed the Widespread Pain Index [37]. The patient is oriented to mark the areas concerning the pain during the last week. Each marked area is equivalent to one point, and the final score varies between 0 and 19 points. Current guidelines recommend the use of the Widespread Pain Index for the identification of generalized pain [37, 38]. Generalized pain is defined as pain in at least 4 of 5 regions (4 quadrants and axial), must be present. The five areas are divided into Region 1 – Left upper region, jaw, shoulder girdle, upper arm, and lower arm (left); Region 2 – Right upper region, jaw, shoulder girdle, upper arm, and lower arm (right); Region 3 – Left lower region, hip, upper leg, and lower leg (left); Region 4 – Right lower region, hip, upper leg, and lower leg (right); and Region 5 – Axial region, neck, upper back, lower back, chest, and abdomen. Jaw, chest, and abdominal pain are not included in generalized pain definition [37]. Widespread Pain Index showed adequate psychometric properties in youth [39].

Conditioned Pain Modulation (CPM) – Cold pressor test is a psychophysical test used to assess the CPM, where the cold pain is the conditioning stimulus, and pressure pain threshold is the test stimulus. The cold pressor test is an appropriate method to assess the descending nociceptive inhibitory system [40] and the most commonly used for the evaluation of CPM [41]. The conditioning stimulus was the immersion of the participants` hand in a bucket with temperature-controlled cold water (1 °C – 4 °C) monitored by a thermometer (5130 model, Incoterm), for up to 1 minute. The participant was instructed to remain with the hand immersed in water without making muscle contractions or changes in position. The withdrawal of the side from the water was allowed when the patient could no longer tolerate the painful stimulus. Room temperature, humidity, lighting, and noise were maintained constant during the entire procedure. Pressure pain threshold was performed before and after 1 minute of the cold pressor test, using a digital pressure algometer (model Force Ten FDX, Wagner Instruments, Greenwich, USA). The distal part of the dorsal forearm and tibialis anterior muscle, which had not been immersed in water, were chosen to be evaluated due to the lack of relationship with participants` musculoskeletal complaints. The two sites were assessed in the same order for all participants. The operation of the pressure algometer and measurement of pressure pain threshold were explained to patients before the assessment. Besides, a familiarization procedure was carried out with the pressure algometer by applying pressure to the dominant forearm to ensure that the test had been understood. The force was gradually increased (1 kg-force/s) until the feeling of pressure from the primary subject was changed to pain. Pressure pain threshold was recorded in kilograms-force (Kgf) when the patient gave the verbal command “pain”. The classification of the efficiency of the CPM was based on the following strategy: evidence of impaired pain modulation in two sites. Only patients with the inefficiency of the CPM in both locations (the anterior tibialis muscle and the distal part of the dorsal forearm) were classified as impaired pain modulation. Upper and lower limb sites were used to avoid the inclusion of the patients with peripheral sensitization according to recommendations for conditioned pain modulation [42]. Also, the efficiency of the CPM was assessed by calculating the difference between the pressure pain threshold values in the cold pressor test (differences between final and initial value). Negative values represented an inefficiency of the CPM, and null or positive values were considered a typical response of the CPM.

Functional limitation – Patient-Specific Functional Scale is a self-reported measure used to assess functional change in patients with musculoskeletal disorders. Patients should identify up to five important activities they are unable to perform or are having difficulty with as a result of their problem and classify on an 11-point scale the current level of difficulty associated with each activity. Patient-Specific Functional Scale has ease applicability and can be used as a clinical outcome measure [43].

### Statistical analysis

The demographic and clinical variables of the study population were summarized as mean (standard deviation) for continuous variables. Categorical variables are presented in absolute (percentage) frequency of the sample. For continuous variables, the normal distribution of the outcomes of the study was verified by the Shapiro-Wilk test. Independent one-way analysis of variance (ANOVA) was used to test for between-group differences (nociceptive pain, unclear, or neuropathic-like symptoms) for the outcome measures with continuous variables (i.e., pain intensity, pain duration, CS-related sign and symptoms, generalized pain, and functional limitation) and Pearson chi-square test (X^2^) verified between-group differences on the efficiency of the conditioned pain modulation. Post-hoc Tukey tests were used for multiple comparisons of means. A significance level of less than 5% (*P* < .05) was considered for all analyses. The statistical analysis was performed using JASP version 0.10.2.0 and Prism for Macintosh, Version 8 (GraphPad Software Inc., San Diego, CA).

## Results

### Characteristics of the participants

A total of 308 patients with musculoskeletal pain with a mean age of 52.21 (±15.01) years old and 220 (71.42%) females was enrolled in this study. One hundred seventy-three (56.16%) participants were classified as nociceptive pain, 69 (22.40%) participants were classified as unclear, and 66 (21.42%) as neuropathic-like symptoms. Generalized pain was described by 33 (19.07%) patients with nociceptive pain, 31 (44.92%) patients classified as unclear, and 33 (50.00%) patients with neuropathic-like symptoms. In addition, 60 (19.48%) participants were classified as impaired CPM. All participants completed the questionnaires and cold pressor test with no adverse events. Then, there were no missing values for the outcomes of the study. The study samples’ characteristics are shown in Table 1.

**Table 1:** Characteristics of the study participants (*n* = 308)

Characteristics	Nociceptive Pain *n* = 173	Unclear *n* = 69	Neuropathic-like symptoms *n* = 66	*p* value
Sex, n (%), female	120 (69.36%)	47 (68.11%)	53 (80.30%)	0.194
Age, mean (SD)	52.26 (15.99)	51.43 (14.72)	52.92 (12.41)	0.846
Weight (kg), mean (SD)	72.83 (16.99)	75.17 (14.08)	72.54 (13.64)	0.546
Height (m), mean (SD)	1.64 (0.11)	1.65 (0.11)	1.60 (0.16)	0.104
Body Mass Index (kg/m^2^), mean (SD)	26.55 (6.60)	28.01 (4.76)	30.86 (25.50)	0.098
Hours of work (weekly), mean (SD)	41.50 (14.75)	45.81 (15.48)	43.60 (17.24)	0.454
Health Insurance (Yes), n (%)	46 (26.59%)	11 (15.94%)	14 (21.21%)	0.170
Physical Exercise (Yes), n (%)	95 (54.91%)	31 (44.92%)	33 (50.00%)	0.425
Final score painDETECT, mean (SD)	6.23 (3.47)c	15.14 (1.49)	23.54 (3.77)a,b	<.001
Pain intensity, mean (SD)	5.26 (2.51)	5.88 (2.14)	7.42 (2.09)a,b	<.001
Pain duration (months), mean (SD)	62.33 (105.78)	59.49 (85.99)	85.58 (100.01)	0.231
CSI, mean (SD)	27.75 (14.26)c	38.53 (14.92)	49.43 (15.94)a,b	<.001
Widespread Pain Index, mean (SD)	3.84 (3.36)c	6.02 (4.26)	8.24 (5.58)a	<.001
Cold Pressor Test, yes, n (%)	32 (18.49%)	15 (21.73%)	13 (19.69%)	0.847
Patient-Specific Functional Scale, mean (SD)	6.77 (2.05)	7.19 (1.81)	8.04 (1.87)a	<.001

Note: Data are presented as mean (SD) for continuous variables and as frequency counts (%) for categorical variables. Significant differences between groups were tested using the unpaired t-test for continuous variables or the chi-square test for categorical variables. ^a^Represents a significant difference between neuropathic-like symptoms group and nociceptive pain group; ^b^Represents a significant difference between neuropathic-like symptoms group and unclear group; ^c^Represents a significant difference between nociceptive pain group and unclear group. Abbreviations: CSI, Central Sensitization Inventory

### Comparison of pain characteristics, CS-related sign and symptoms, generalized pain, functional limitation, and conditioned pain modulation

A ANOVA showed differences for the pain intensity [F (2, 305) = 20.097; *p* < .001], Central Sensitization-related sign and symptoms [F (2, 305) = 54.186; *p* < .001], pain area [F (2, 305) = 28.525; *p* < .001], and functional limitation [F (2, 256) = 8.061; *p* < .001]. CPM was similarly impaired among the three groups (*X*^2^ = 0.333, *p* = 0.847).

## Discussion

This study compared the pain characteristics of patients with musculoskeletal pain classified as nociceptive pain, unclear, and neuropathic-like symptoms according to the painDETECT questionnaire. Our sample demonstrated similar demographic and clinical features among the groups but different pain phenotypes. Patients with neuropathic-like symptoms had more pronounced pain intensity, higher levels of CS-related signs and symptoms, and presented more generalized pain than patients classified as nociceptive pain, confirming our hypothesis. CPM demonstrated to be similar among the three groups. Besides, the functionally was more restricted in patients with neuropathic-like symptoms than patients with nociceptive and unclear groups.

The current findings revealed that the painDETECT was able to identify different musculoskeletal pain phenotypes. Identifying those phenotypes can have relevance for clinical practice and aid in developing of adequate interventions for the treatment of patients with musculoskeletal disorders [5]. Furthermore, the assessment of phenotypes has been performed in previous studies with patients presenting musculoskeletal pain. Patients with knee osteoarthritis classified as neuropathic-like symptoms in painDETECT present increased pain, widespread, and impaired physical function compared with other groups [44]. The authors concluded that the recognition of knee osteoarthritis patients with this phenotype can offer of targeted and effective approaches [44]. Furthermore, chronic low back pain patients with neuropathic-like symptoms reported more severe pain, poorer physical and mental health, exhibited higher back pain-related disability, signs of depression, and psychological distress when compared to chronic low back pain patients classified as nociceptive pain [12]. Ultimately, painDETECT detected distinct clinical profiles for chronic low back pain patients.

Our findings revealed that patients classified as neuropathic-like symptoms had a higher number of pain areas and greater levels of symptoms of central sensitization than patients with nociceptive or unclear classification. Bilateral sensory abnormalities were found in patients with unilateral neuropathic pain [45], and patients with peripheral neuropathies had minimal sensory differences between the affected and non-affected area [46]. Patients with neuropathic-like knee pain showed meaningful association with widespread pain compared with other patients [47]. Similarly, patients with unilateral carpal tunnel syndrome had bilateral thermal hyperalgesia, supporting the role of generalized sensitization mechanisms of pain [48]. Widespread pain and central sensitization also have been related [17, 49–52], and there is clinical evidence that central sensitization is present in patients with neuropathic pain [19]. Furthermore, widespread sensitization has been described in many musculoskeletal conditions (i.e., migraine and chronic tension-type headache [53], painful knee osteoarthritis [54], and unilateral epicondylitis [55]). Thus, our findings highlight the relationship between generalized pain and symptoms of central sensitization in patients with neuropathic-like symptoms.

Several approaches are available to assess central sensitization. CSI is a clinically useful prediction tool regardless of patients with musculoskeletal disorders [56]. Another strategy for assessing central sensitization is CPM through the cold pressor test that evaluates the descending nociceptive inhibitory system. Our findings revealed that patients classified with neuropathic-like symptoms present higher scores in CSI. This result is consistent with previous studies showing that central sensitization is most manifested in painful conditions with the neuropathic component [16, 17]. However, the current study showed that 19% of patients classified as neuropathic-like symptoms presented impaired CPM in the cold pressor test, revealing that the patient who presents more Central Sensitization-related sign and symptoms is not related to the impaired CPM. Besides, CSI scores were not associated with the efficiency of the CPM [18, 57], and CSI had limited applicability for detecting the deficit in the CPM in patients with chronic musculoskeletal pain [58]. The adequate CSI measurement properties may be related to a particular subgroup of patients with psychosocial aspects [58], considering the CSI scores are associated with anxiety and depression rather than psychophysical measures of central sensitization [57, 59].

We acknowledge the strengths and limitations of the present study. First, this is the first study to compare the pain characteristics of patients with nociceptive, unclear and neuropathic-like symptoms altogether. Second, painDETECT is a well-recognized screening tool for the identification of neuropathic-like symptoms [60]. Finally, the large sample size can be considered as a strength in this study. Regarding study’s limitation, first is the lack of a health condition diagnosis which may affect the findings in particular condition. Second, the cold pressor testis not gold-standard for the diagnosis of the impaired CPM. Nevertheless, cold pressor test is the most common method used for CPM assessment [41]. Ultimately, the current study is a cross-sectional design which limits the generalizability of the findings. However, we adopted a multicenter design and implemented many methods to minimize the risk of bias following current guidelines for this type of study.

Our study provides new insight for implementation of the painDETECT in clinical use and further studies. PainDETECT is a low-cost and simple screening instrument for assessing and identifying neuropathic-like symptoms and should be implemented for evaluating pain phenotypes in patients with heterogeneous musculoskeletal pain. The identification of pain features of patients with musculoskeletal pain contributes to tailored treatment. Thus, health professionals are encouraged to incorporate in their practice strategies for the management of patients with musculoskeletal pain according to their pain predominance (i.e., nociceptive or neuropathic). Clinicians should be aware of the more severe pain characteristics of patients with a neuropathic component. Future studies in different populations and settings are necessary to compare the features of musculoskeletal pain according to their predominance.

## Conclusion

PainDETECT identifies different musculoskeletal pain phenotypes. Patients with neuropathic-like symptoms revealed unfavorable pain characteristics compared to their counterparts, including pain intensity, CS-related sign and symptoms, generalized pain, and functional limitation.

## Data Availability

Not applicable.
